# Development of a Digital Olfactory Function Test: A Preliminary Study

**DOI:** 10.3390/life14010075

**Published:** 2024-01-02

**Authors:** Hae Ryong Lee, Kyung Soo Kim, Hyun Jin Min

**Affiliations:** 1 Research and Development Center, Digital Scent Co., Ltd., Daejeon 305-700, Republic of Korea; skybandpeter@daum.net; 2 Department of Otorhinolaryngology-Head and Neck Surgery, Chung-Ang University College of Medicine, 224-1 Heukseok-dong, Dongjak-gu, Seoul 06973, Republic of Korea

**Keywords:** digital olfactory test, olfaction, psychophysical testing, threshold–discrimination–identification (TDI)

## Abstract

Olfactory dysfunction is associated with conditions such as neurodegenerative diseases, diabetes, obesity, autoimmune diseases, mental illnesses, and upper-airway-related diseases. The COVID-19 pandemic necessitated the development of an examiner-independent olfactory function test. We recently developed a digital olfactory function test called Digitalscent (DIGITAL SCENT), which is a kiosk-type device with an integrated hardware system. The protocol follows conventional psychophysical olfactory function protocols, including threshold, discrimination, and identification test subsets. Eight healthy participants without olfactory dysfunction volunteered for the suitability test and completed both the YSK olfactory function and Digitalscent tests. Pearson correlations were determined between the YSK olfactory function and Digitalscent tests. Digitalscent could be implemented as a conventional olfactory function test, and all participants followed the Digitalscent test protocols. Limitations in the threshold and identification test subsets included unfamiliarity of the patients to the digital test and incompleteness in the sophisticated release of odorants. A strength of the identification test subset was the dual sensory stimulation of vision and olfaction. Digitalscent could—without facilitating viral transmission—enable early diagnosis of olfactory dysfunction during respiratory viral pandemics. Future studies with larger sample sizes are warranted to facilitate wider use of this digital olfactory function test.

## 1. Introduction

Olfaction is the ability to detect odors, which is helpful in detecting hazards such as spoiled food, gas leaks, and fires [1,2]. Therefore, olfaction is a determinant of quality of life and is crucial for survival. Olfactory dysfunction (OD) may occur during the natural aging process or after unexpected events, such as viral infections or head trauma. OD could also accompany neurodegenerative diseases as an early sign or during the progression of diseases, such as Alzheimer’s or Parkinson’s disease [3]. Symptoms related to OD have been more commonly encountered since the onset of the COVID-19 pandemic, and the significance of the appropriate diagnosis of OD has been emphasized in ear, nose, and throat departments.

Various psychophysical olfactory function tests have been developed and applied in clinical practice. These tests are usually based on Sniffin’ sticks testing and are composed of odorant detection threshold, discrimination, and identification subsets [4]. As previous investigations have reported a lack of an association between the results of psychophysical testing and patients’ subjective reports, the results of these olfactory function tests are essential in the diagnosis and management of olfactory dysfunction [5]. These psychophysical tests pose some challenges, including considerable variations in the reliability between tests, the subjectivity of individual responses, lack of time on the part of the investigator, and the degree of agreement among independent examiners [6]. Among these, the standardization of tests and the training of examiners are important issues that impact the reliability of the test results [7]. Furthermore, the COVID-19 pandemic necessitated the development of an examiner-independent olfactory function test for use at symptom onset without the risk of viral spread.

There have been trials to digitize the measurement of olfactory function. For example, Nakanishi et al. introduced a digital scent device that enables the measurement of identification test scores using 40 odors [6]. A mobile digital olfactory testing system consisting of an identification test has also been introduced [8]. We aimed to develop a digital olfactory function test that mimics conventional psychophysical olfactory function tests with obligatory threshold, discrimination, and identification subsets. The systematization of olfactory measurements is necessary for the development of a digital olfactory function test. The stable storage of odorants, sophisticated control of odorant delivery, and digitization of all the processes of the test and recording of the results are thought to be major hurdles in the development of an olfactory function test [6]. Recently, we have succeeded in digitizing all the processes of a conventional psychophysical olfactory function test composed of threshold, discrimination, and identification subsets, and we performed a preliminary clinical study to evaluate its suitability. In this study, we aimed to introduce the outcomes of our development, called “Digitalscent,” and present the results of our preliminary suitability evaluation.

## 2. Materials and Methods

### 2.1. Participants and Study Design

This was a single-center study conducted at Chung-Ang University Hospital, which is a tertiary medical center, and all the study protocols were approved by the Institutional Review Board of the Chung-Ang University Hospital (2208-019-520). Informed consent was obtained from all enrolled participants. As this study was a preliminary study to evaluate the suitability of the Digitalscent test during its development, we limited the number of participants and included specialized rhinology doctors who could provide feedback about the test. Eight volunteers were enrolled in this study: two rhinology specialists, two nurses, and four staff members outside the clinical environment (secretaries and computer technicians). None of the enrolled participants had any subjective OD symptoms. We excluded individuals with paranasal sinus diseases (detected using nasal endoscopy), including upper airway infectious symptoms, metabolic disorders, and head trauma, as well as those with neurodegenerative diseases that might affect the test results. All enrolled participants underwent the YSK olfactory function test and the Digitalscent olfactory function test on the same day, with a 1 h interval between the tests.

### 2.2. YSK Olfactory Function Test

The YSK olfactory function test (RHICO Medical Co., Seoul, Republic of Korea) is a validated, conventional psychophysical olfactory function test composed of three subsets: threshold, discrimination, and identification [1]. The YSK olfactory function test was performed by a skilled examiner according to the manufacturer’s instructions. The detection threshold was defined as the concentration at which 2-phenylethyl alcohol (PEA) was correctly identified four consecutive times (highest concentration: 10%; 1:2 serial dilutions for 12 steps). The clinician switched from the lowest concentration (level-12 pen) to the level-10 pen, then continued to switch to a pen two levels higher until the subject correctly identified the odorant. If the subject was correct twice consecutively, this became the first inflection point, and the concentration was reduced by one level. This time, if the subject was correct twice consecutively, the concentration was reduced by another level. However, if the subject was incorrect even once, this level was the second inflection point. In this way, for finding the odd-numbered pen levels, the inflection point moved up by two levels until the subject correctly identified the odorant twice consecutively. When looking for even-numbered inflection points, the concentration was sequentially reduced by one level until the subject was incorrect. The mean score of the last four inflection points out of a total of seven inflection points was the olfactory threshold score [1]. The discrimination score was defined as the number of correct answers when choosing the different odorant among three odorants (2 identical, 1 different). The subject smelled all three test pens and then selected a target odorant that smelled different from the other two. The total number of correct answers among the 12 steps was summed and evaluated [1]. The identification score was defined as the number of correct answers based on multiple forced choices from four descriptors. The threshold–discrimination–identification (TDI) score was the sum of the obtained threshold, discrimination, and identification scores, ranging from 1 to 36. The diagnostic cut-off for anosmia was a TDI score ≤ 14.5, and that for hyposmia was 14.5 < TDI ≤ 21.0. All test procedures were performed in a well-ventilated laboratory.

### 2.3. Digitalscent Olfactory Function Test

**Figure 1:**
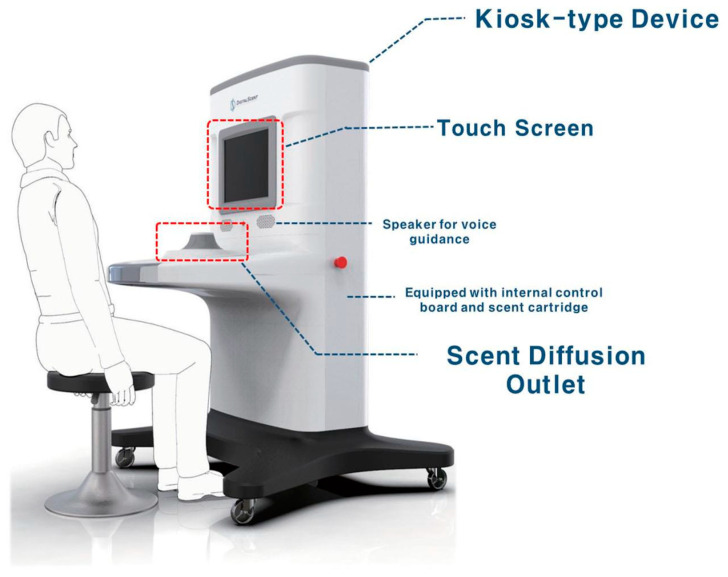
The Digitalscent olfactory function test device.

The Digitalscent olfactory function test device (DIGITAL SCENT CO., Ltd., Daejeon, Republic of Korea) was developed as a kiosk-type device with an integrated hardware system comprising a central processing unit, touchscreen, Wi-Fi antenna, Bluetooth interface, USB dock, power supply, and odor system. A total of 26 scent cartridges were installed in an odor system, 10 of which contained PEA, a scent for threshold testing, at 10 concentration levels (highest concentration: 10%; 1:2 serial dilutions for 10 steps) in the form of a liquid scent (one concentration level per cartridge). We applied the same odorants used in YSK olfactory function testing for the threshold and discrimination test subsets. The remaining 16 scents (rose, apple, watermelon, coffee, strawberry, soy sauce, red ginseng, sweet potato, pine needles, sesame oil, green grape, cinnamon, acacia, grass, peppermint, and scorched rice) [9] were used for the identification tests. Each scent contained in these cartridges was emitted to the discharge port via pumps and valves controlled by a processor so that the corresponding scent was accurately emitted (Figure 1).

Figure 1 depicts the digital olfactory function test device. The device’s user interface sequentially shows the login screen, menu screen, olfactory testing guidance screen, olfaction guidance screen, threshold test item screen, threshold test result screen, identification test item screen, identification test result screen, discrimination test item screen, and discrimination test result screen.

**Figure 2:**
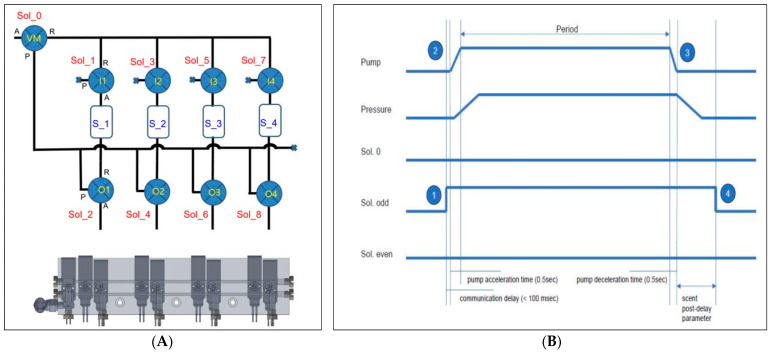
Scent diffusion process. (**A**) Carrier gas line diagram for scent diffusion and cleaning. Scent diffusion is controlled by a direction-switching valve to pass through the source, while cleaning is carried out by bypassing the source. Cleaning is performed after scent diffusion to remove any remaining residue in the diffusion value line. (**B**) Timing chart for pump and valve control during the scent diffusion process. Scent diffusion begins with the control of the direction-switching valve in the source line (➀) to prevent mixing of the scent. The pump is then started (➁), leading to an increase in pressure, which initiates the flow of the carrier gas and the diffusion process through the source. The diffusion process concludes with the stopping of the pump (➂), followed by waiting until the flow of carrier gas ceases, and then controlling the direction-switching valve (➃).

The digital olfactory function test device hardware consists of a microcontroller-based control unit that allows the user to regulate the emission of odors and air through controlling a pump. The digital olfactory function test device hardware contains a small processor for independent olfactory testing, requiring a separate software execution platform. Figure 2 depicts the controlling algorithm, wherein the main processor sends a command to the solenoid valve for the device to turn on, receive responses, and operate the pump. The board that controls the solenoid valve operates a timer to turn off the solenoid valve after a certain period of time has passed.

The digital olfactory function test device software runs on a computing device equipped with an operating system. The software manages a series of procedures to emit the necessary odors for testing, remove lingering scents, control concentrations, and compose related commands as messages to be conveyed to the digital olfactory function test device hardware, directing it to perform olfactory testing.

There are three types of tests: olfactory threshold, olfactory identification, and olfactory discrimination tests. The olfactory threshold test measures the ability to smell through adjusting the concentration of odors such as PEA (2-phenylethyl alcohol) from low to high concentrations. During three odor presentations, two are odorless, and one contains PEA. The subject must identify the sequence of PEA presentation versus odorlessness. If the subject is incorrect, the odor concentration increases by two levels; if the subject is correct, it decreases by one level. This process is repeated, and a graph is plotted to find seven inflection points. The mean of the last four inflection points determines the olfactory ability.

The purpose of the olfactory identification test is to determine whether the brain can process information about smell through testing whether the user can be reminded of the name when smelling. To do this, a scent is presented, and scent name candidates are presented in multiple-choice format for the user to select. Across three odor presentations, two are the same, and one is different. The participant must identify the different odor.

The purpose of the olfactory discrimination test is to evaluate whether the user’s sense of smell can recognize and distinguish differences in odors. To this end, the user is sequentially sprayed with two whiffs of the same scent and one different scent and asked to indicate the different scent.

**Figure 3:**
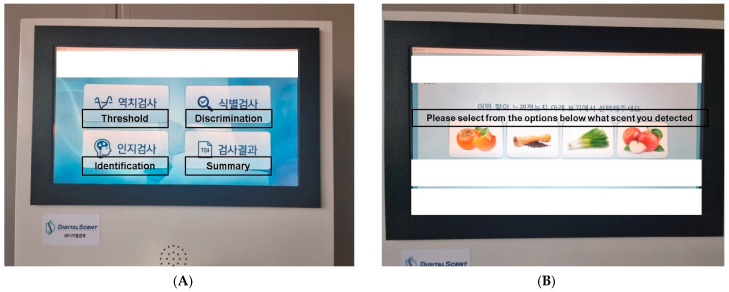
Digitalscent screen images. (**A**) The Digitalscent screen is operated through touch gestures without tactile buttons. (**B**) Screen image of the identification subset test, which enables selections based on images.

For each selected test, the purpose and procedure are visually or verbally explained. Scores are calculated after test completion. In the olfactory threshold test, the score is the mean of the last four inflection points. For the olfactory identification and discrimination tests, the score is the accuracy rate across all tests. If the test is conducted via a touchscreen, on-screen instructions guide the participant through the process, facilitated via touch buttons. When user responses are required, choices are provided on the screen (Figure 3).

The olfactory device selects the required odor for testing through choosing the corresponding cartridge and configuring parameters to compose olfaction command messages. The piping device manages post-olfaction cleaning via composing command messages to remove lingering scents, conveying these commands from the software to the olfactory device hardware. After the entire testing process is complete, the TDI score is calculated as the summation of the threshold, discrimination, and identification test scores.

### 2.4. Statistical Analysis

All continuous variables are reported as the mean ± standard deviation. Pearson correlation coefficients were calculated to determine the correlations between the measurement outcomes and the YSK olfactory function and Digitalscent tests. All statistical analyses were performed using GraphPad Prism, version 8.0 (GraphPad Software, San Diego, CA, USA). *p*-values less than 0.05 were considered statistically significant for all analyses.

## 3. Results

The demographic data of the participants and the results of the olfactory function tests are summarized in Table 1. Among the eight volunteers, three were men, and five were women; the mean age was 41.12 ± 10.53 years. None of the participants had medical conditions, such as hypertension or diabetes mellitus. The mean threshold score obtained using the YSK olfactory function test was 5.85 ± 1.99. The mean discrimination score was 9.00 ± 1.07, and the mean identification score was 11.75 ± 0.46. The mean TDI score was 26.37 ± 11.0, and all the participants were identified as normosmic based on the YSK olfactory function test results. The mean threshold score obtained using the Digitalscent test was 8.06 ± 4.03. The mean discrimination score was 4.73 ± 1.82, and the mean identification score was 10.53 ± 1.50. The mean TDI score obtained using the Digitalscent test was 23.75 ± 5.12 (Table 1).

**Figure 4:**
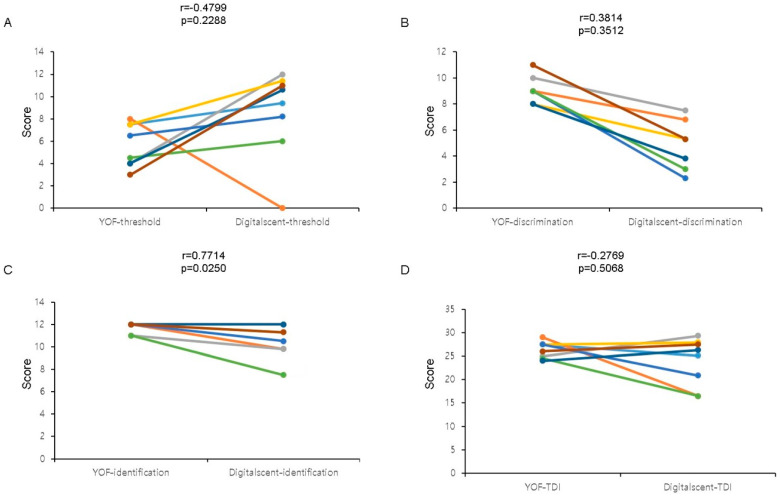
The linear correlation between the Digitalscent and the YSK olfactory function tests. (**A**) Threshold test scores, (**B**) discrimination test scores, (**C**) identification test scores, and (**D**) TDI (overall threshold-discrimination-identification) scores were compared. The *p*-values < 0.05 were considered statistically significant.

A comparison of the subsets of the YSK olfactory function and Digitalscent tests revealed that although there was no significant correlation between the threshold and discrimination test subsets (Figure 4A,B), there was a significant correlation between the identification test subset results (Pearson coefficient = 0.7714, *p* = 0.0250) (Figure 4C). There was no significant correlation between the TDI scores of the two tests (Figure 4D). Based on the r-values, there were negative correlations between the YSK olfactory function test scores and the Digitalscent threshold (r = −0.4799) and TDI (r = −0.2769) scores, respectively (Figure 4A,D).

**Figure 5:**
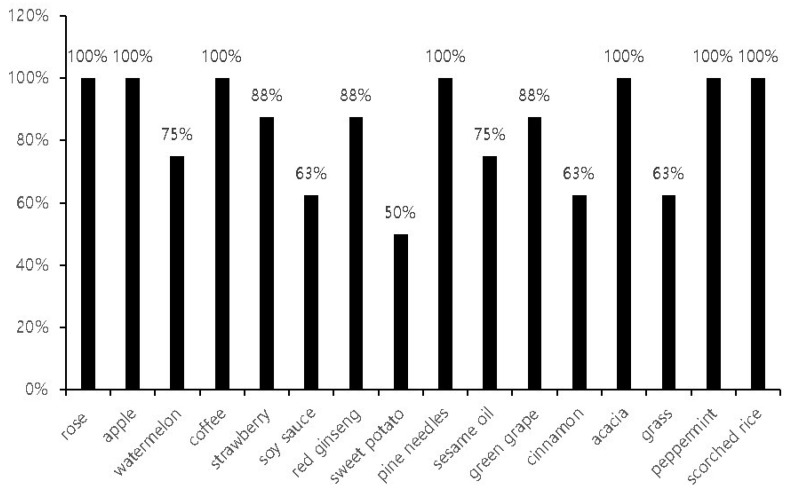
The correct odorant identification rates according to the odorants in the Digitalscent identification subset.

In the next step, we further investigated the measurement outcomes of the identification tests. We found that the mean rate of correct responses during the identification subset of the Digitalscent test was 84 ± 17%. All participants chose correct responses for the rose, apple, coffee, pine, acacia, peppermint, and scorched rice odorants (correct answer rate = 100%). In contrast, the mean correct answer rates were lower for the sauce, cinnamon, grass, and sweet potato odorants, with only about 60% of the participants responding correctly (Figure 5).

## 4. Discussion

We developed our digital, self-administered olfactory function test, Digitalscent, reflecting the protocols of a conventional psychophysical olfactory function test. Studies demonstrating or investigating computer-based, digital, olfactory function tests that reproduce all subsets (threshold, discrimination, and identification) of a psychophysical olfactory assessment without an examiner are rare. Although we found a report of a digital scent device for olfactory assessment [6], it was limited to identification testing and did not involve threshold and discrimination tests.

To facilitate successful digital olfactory function testing, it is important to develop software that allows the odorant to be attached to the device, the odor source to be delivered to the patient’s nose through adjusting the odor, and the participant to be connected to the following procedure according to their response. Through this preliminary study, we confirmed that Digitalscent implemented a series of conventional olfactory function tests, starting from threshold testing and sequentially implementing discrimination and identification tests, to yield the final results.

We found that there might be some difficulty in the threshold subset testing for some individuals. In the present study, one participant failed to detect odorants during the threshold test, while another participant obtained relatively low scores in the threshold test relative to the other subsets (Figure 4). Test subjects, especially older adults, may not be familiar with a fully digital test procedure. Since the threshold test is the first to be performed during the test, this unfamiliarity could be one of the reasons for the above-mentioned difficulties. Additionally, problems—such as odorant mixing—with the sophisticated serial release of odorants with increasing concentrations could also contribute to these challenges. Therefore, we inferred that more detailed information must be given about the test before beginning and that the odorant-releasing program must be technically adjusted.

During discrimination testing, the scores obtained with the Digitalscent test were lower than those obtained with the YSK olfactory function test (Figure 4B). The discrimination subset requires subjects to distinguish between odorants. Our discrimination test protocol was designed to require subjects to identify three different sequentially released odorants. If a discrimination test is manually performed by an examiner, the participant could ask for another chance to detect the first odorant before identifying it. However, our protocol did not provide participants with another chance to smell the first odorant, the perception of which could have faded after the other odorants were smelled. This could be the reason for the lower scores associated with the Digitalscent discrimination testing relative to the corresponding YSK olfactory function test subset, and we are considering allowing users to re-smell previously released odorants. After reviewing the threshold and discrimination test processes, we are planning to re-check and modify the current system to prevent odorant mixing and improve score calculations.

The Digitalscent identification test is composed of a dual-sensory combination of visual–olfactory stimulation. During the Digitalscent identification test, four different odorants were released, and the participants were asked to identify the odorants through touching the respective corresponding images on a touchscreen (Figure 3). This combined visual–olfactory measurement tool proved to be a strength of Digitalscent during the identification test. The Digitalscent identification test results correlated significantly with those of the YSK olfactory function test, and the raw scores of both tests were similarly distributed. Similarly, with the YSK olfactory function test, the scores from the identification subset in the normosmia range were significantly higher than those from the other subsets, suggesting the significance of selecting culture-specific odorants in conventional olfactory function testing [1]. Additionally, the verbal identification of odors, as in conventional psychophysical olfactory function testing, is affected by conditions affecting the brain, such as trauma, without any definite olfactory impairment [10]. Therefore, choosing the corresponding images representing the specific odorants through touching the images shown on a screen might be beneficial to those patients with speech impairments. Furthermore, the efficacy of olfactory training in patients with COVID-19-associated olfactory loss was shown to be the highest when visual and olfactory stimulation were combined [11]. Olfactory training works on improving the subject’s sense of smell via facilitating practice and training of the olfactory system via the repeated smelling of various scents. We suggest that well-controlled repeated release of various odorants from the device could be applied for olfactory training. Therefore, the possibility of using Digitalscent’s identification test (which uses both visual and olfactory sensory stimulation) as an olfactory training tool should be evaluated in future studies.

Finally, a substantial benefit of this novel olfactory testing system is the ability to use different odorants within the same system. Cognitive factors have significant impacts on tasks assessing odor identification abilities, and odor identification requires knowledge of the specific odors [12]. Therefore, odorants used in identification tests should be culture-specific. The application employed in our system uses interchangeable cartridges; the same hardware and software can be adopted in other countries through using culture-specific odorant cartridges.

A limitation of this study was the small sample size. Unsurprisingly, all participants were normosmic. Therefore, our study did not confirm the validity or reliability of Digitalscent compared with other tests. Instead, this was a preliminary study to evaluate the suitability of a fully digital, computer-based, olfactory function test that mimics conventional psychophysical testing. We found that Digitalscent allowed for the implementation of odor threshold, discrimination, and identification tests without an examiner. We plan to validate these results through conducting larger-scale research and comparing the results with those yielded using previously developed conventional olfactory function tests. We also identified some limitations in the threshold and discrimination test subsets; future studies will focus on overcoming such limitations.

## 5. Conclusions

Digitalscent is a digital, computer-based psychophysical olfactory function test consisting of threshold, discrimination, and identification subsets. In this preliminary suitability study, we identified some limitations and strengths of Digitalscent. Future studies are needed to further develop the current Digitalscent testing system and investigate the reliability and normal values of the test. These trials might improve the feasibility of examiner-free olfactory function testing, which would be of great benefit, especially in the context of avoiding viral spread during a respiratory virus pandemic.

## Figures and Tables

**Table 1:** Demographic and clinical characteristics of the enrolled participants.

Variable	Value
Participants (N)	8
Sex (M/F)	3/5
Age (years) (mean ± SD)	41.12 ± 10.53
YSK olfactory function test	
Threshold score (mean ± SD)	5.85 ± 1.99
Discrimination score (mean ± SD)	9.00 ± 1.07
Identification score (mean ± SD)	11.75 ± 0.46
TDI score (mean ± SD)	26.37 ± 11.0
Digitalscent test	
Threshold score (mean ± SD)	8.06 ± 4.03
Discrimination score (mean ± SD)	4.73 ± 1.82
Identification score (mean ± SD)	10.53 ± 1.50
TDI score (mean ± SD)	23.75 ± 5.12

M, male; F, female; TDI, threshold–discrimination–identification; SD, standard deviation.

## Data Availability

Data is contained within the article and Supplementary Material. Data from this study are available from the corresponding author upon reasonable request. The data are not publicly available.

## Supplementary Materials

The following supporting information can be downloaded at: https://www.mdpi.com/article/10.3390/life14010075/s1.
